# Identification of ST3GAL5 as a prognostic biomarker correlating with CD8^+^ T cell exhaustion in clear cell renal cell carcinoma

**DOI:** 10.3389/fimmu.2022.979605

**Published:** 2022-09-12

**Authors:** Jiakuan Liu, Meiqian Li, Jiajun Wu, Qi Qi, Yang Li, Simei Wang, Shengjie Liang, Yuqing Zhang, Zhitao Zhu, Ruimin Huang, Jun Yan, Rujian Zhu

**Affiliations:** ^1^ Department of Urology, Shanghai Pudong Hospital, Fudan University Pudong Medical Center, Shanghai, China; ^2^ Department of Laboratory Animal Science, Fudan University, Shanghai, China; ^3^ Model Animal Research Center of Nanjing University, Nanjing University, Jiangsu, China; ^4^ Shanghai Institute of Materia Medica, Chinese Academy of Sciences, Shanghai, China; ^5^ University of Chinese Academy of Sciences, Beijing, China

**Keywords:** sialylation, clear cell renal cell carcinoma, ST3GAL5, CD8^+^ T cell exhaustion, prognostic biomarker

## Abstract

Aberrant sialylation is frequently observed in tumor development, but which sialyltransferases are involved in this event are not well known. Herein, we performed comprehensive analyses on six ST3GAL family members, the α-2,3 sialyltransferases, in clear cell renal cell carcinoma (ccRCC) from public datasets. Only ST3GAL5 was consistently and significantly overexpressed in ccRCC (n = 791 in total), compared with normal kidney tissues. Its overexpression was positively correlated with tumor stage, grade, and the poor prognosis in ccRCC patients. Gene Ontology and Kyoto Encyclopedia of Genes and Genomes pathway enrichment analyses indicated the involvement of ST3GAL5 in tumor immunoregulation. Then we revealed that ST3GAL5 expression showed a positive correlation with CD8^+^ T cell infiltration, using multiple tools on TIMER2.0 web server. Notably, ST3GAL5 overexpression was further identified to be associated with expression signature of CD8^+^ T cell exhaustion in ccRCC samples from three datasets (n = 867 in total; r > 0.3, p < 0.001). In our own ccRCC cohort (n = 45), immunohistochemistry and immunofluorescence staining confirmed that ST3GAL5 overexpression was accompanied by high CD8^+^ T cell infiltration with the increased exhaustion markers. Altogether, ST3GAL5 as a promising prognostic biomarker with CD8^+^ T cell exhaustion in ccRCC is indicated.

## Introduction

Kidney cancer is one of the common malignancies in urologic system. Approximately 430,000 individuals are diagnosed annually with kidney cancer and over 179,000 patients die of it worldwide (1). Clear cell renal cell carcinoma (ccRCC) accounts for ~70% of kidney cancer cases and the majority of kidney cancer-related deaths (1–3). Though localized ccRCC can be treated with surgery, about 30% ccRCC patients will relapse and develop metastasis, which is lethal with a 5-year survival rate of less than 15% (2, 3). Single-cell RNA sequencing reveals enrichment of terminal exhausted CD8^+^ T cells in advanced ccRCC, indicating that immune dysfunction has become an attractive characteristic of advanced ccRCC (4).

Sialylation is an important glycosylation modification and involved in many biological processes, including cell-cell recognition and adhesion (5). Aberrant sialylation on cell surface is considered as a malignant feature of cancer cells, mediating tumor cells survival, invasion, migration and immune evasion (6–8). In many cancers, high level of sialylation on the surface of cancer cells is associated with high malignant potential and poor clinical outcome of patients, indicating that sialic acid is a potential therapeutic target (9). Many studies show that the abnormal high levels or activities of sialyltransferases are associated with the progression of several tumors (10). In breast cancer, higher serum sialyltransferases levels and activities were strongly linked with higher tumor stage, which enabled them as the biomarkers for monitoring tumor progression and therapeutic effect (11).

Sialyltransferases are divided into four subfamilies: α-2,3 sialyltransferases (ST3GAL1-6), α-2,6 sialyltransferases (ST6GAL1-2 and ST6GALNAC1-6), and α-2,8 sialyltransferases (ST8SIA1-6) (5). The increased level of α-2,3-sialylated glycan was implicated in chemoresistance of cholangiocarcinoma cells to 5-FU and glioma stemness maintenance (12, 13). Moreover, α-2,3-sialylated prostate-specific antigen (PSA) could serve as a better diagnostic marker than conventional ones (total PSA or PSA density) in prostate cancer patients (14). Correspondingly, ST3GAL family members have been reported to be involved in the progression of many cancers and become the potential diagnostic and/or prognostic biomarkers in certain cancer patients (15–17). For example, ST3GAL1 could be a promising therapeutic target in melanoma owing to its role in promoting tumor metastasis (18). ST3GAL3, 4 and 6 increased the biosynthesis of sialyl Lewis x (SLe^x^) antigen, an important tumor-associated antigen associated with poor survival in cancer patients (19, 20). ST3GAL6 was identified as a potential biomarker to predict clinical outcomes in bladder cancer patients, and its overexpression was associated with basal subtype and required for cancer cells invasion (17). Moreover, ST3GAL1 promoted tumor immune evasion by mediating the sialylation of CD55 (21).

In this study, six ST3GAL family members were investigated by comprehensive analysis in ccRCCs using their expressional levels from multiple public datasets. ST3GAL5 overexpression was identified to successfully predict poor prognosis and CD8^+^ T cell exhaustion in ccRCC, which was further validated in our own cohort using immunohistochemistry and immunofluorescence staining methods.

## Materials and methods

### Differential expression analyses in ccRCC on Oncomine platform

The mRNA levels of ST3GAL family members in ccRCC samples from five publish data (Jones et al. (2005) (22); Gumz et al. (2007) (23); Beroukhim et al. (2009) (24); Higgins et al. (2003) (25); Yusenko et al. (2009) (26)) were analyzed by online Oncomine tool (https://www.oncomine.org). The parameters were set as follows, data type: mRNA expression; analysis type: cancer vs. normal; gene rank = all; fold change ≥ 1.5; p < 0.05, using Student’s *t*-test.

### Data source

The data for gene expression and clinical information were downloaded from NCBI GEO database (GSE66272 (27), GSE53757 (28), GSE36895 (29), GSE105261 (30) and GSE73731 (31)), TCGA-KIRC (https://portal.gdc.cancer.gov/), and cBioPortal (https://www.cbioportal.org/). CD8^+^ immunofluorescence intensities and RNA-sequencing data were obtained from Braun et al. (2020) (32).

### Kaplan-Meier survival analysis

The samples were evenly divided into low and high expression groups, according to mRNA level of ST3GAL family members or immune score by xCell algorithm from TIMER2.0 (https://www.timer.cistrome.org/) (33). Kaplan-Meier survival curves with log-rank test were applied to analyze overall survival (OS) and disease-free survival (DFS) in ccRCC patients. p < 0.05 was considered as statistically significant. The significance of gene relationship with OS/DFS was measured by hazard ratio (HR) and 95% confidence interval (CI) in Cox Proportional-Hazards Model. HR > 1, was considered as an increased risk.

### Gene ontology and Kyoto encyclopedia of genes and genomes pathway enrichment analyses

The co-expressed genes with ST3GAL5 in TCGA-KIRC dataset were identified by Spearman’s correlation analysis from cBioPortal (https://www.cbioportal.org/) using r ≥ 0.3 as a cut-off value. GO and KEGG pathway enrichment analyses were performed respectively using DAVID online tool (https://www.david.ncifcrf.gov/). According to the p-valve list, the top 30 enriched GO terms, including 10 biological processes (BP), 10 cellular components (CC) and 10 molecular function (MF) categories, as well as 15 KEGG pathways were presented by bubble diagrams to show their relations to *ST3GAL5* gene expression.

### Analyses by TIMER2.0 web server

The relative values of immune score and infiltration estimation fraction of immune cells for TCGA-KIRC were obtained by xCell algorithm from TIMER2.0 (http://timer.cistrome.org). Immune score is an experimental score obtained by dividing the sum of these infiltration estimation fraction of B cell, CD4^+^ T cell, CD8^+^ T cell, eosinophil, macrophage, mast cell, monocyte, neutrophil, NK cell and myeloid dendritic cell by 1.5. Heatmaps were plotted using FPKM of *ST3GAL5* gene, immune score and infiltration estimation fraction by Cluster 3.0 software (34).

To evaluate the correlation of *ST3GAL5* expression with CD8^+^ T cell infiltration in TCGA-KIRC, xCell, MCP-counter, quanTIseq, and EPIC algorithms were respectively used. In brief, the gene name (*ST3GAL5*) and T cell CD8^+^ were input in the “Immune Association” module to assess their correlation by Spearman analysis using Rho value. Scores of CD8^+^ T cell infiltration were adjusted by cell purity for xCell and MCP-counter algorithms; no adjustment for CD8^+^ T cell infiltration score was required for quanTIseq and EPIC algorithms.

### Exhaustion signature score for CD8^+^T cells

The genes used for CD8^+^ T cell exhaustion signature were obtained from Kfoury et al. (2021) (35) and listed in **Table S1**.The CD8^+^ T cell exhaustion signature scores were then calculated using Gene Set Variation analysis (GSVA) in R package. GSVA was used to estimate variation of gene set enrichment over samples population in a non-parametric and unsupervised manner.

### Clinical samples

Forty-five ccRCC samples were from Shanghai Pudong Hospital (Shanghai, China) with the approval by the Ethics Committee of Shanghai Pudong Hospital and the written informed consents of corresponding patients.

### Immunohistochemistry and immunofluorescence staining

IHC staining on paraffin-embedded sections performed as described previously (36). Briefly, ccRCC paraffin sections were deparaffinized, antigen retrieval in citrate buffer (pH 6.0), blocked, incubated respectively with anti-ST3GAL5 (1:1,000; 14614-1-AP; Proteintech, Rosemont, IL), anti-CD8 (1:5,000; 66868-1-Ig; Proteintech) and anti-PD-1 (1:5,000; 18106-1-AP; Proteintech), and visualized by DAB detection kit (DAB-2031; MXB Biotechnologies, Fuzhou, China). Normal rabbit IgG (KIT-9707; MXB Biotechnologies) and mouse IgG (KIT-9701; MXB Biotechnologies) were used as negative controls. Images were acquired by a slide scanner (NanoZoomer 2.0-HT; HAMMATSU, Japan) and analyzed by NDP serve slide distribution and management software (HAMMATSU). The IHC score was the multiplication of the intensity value (0-3) and the positive ratio value (0-3) of ST3GAL5 immunoreactive cells. The number of PD-1 and CD8 positive cells were enumerated.

For IF staining, sections were deparaffinized, antigen retrieval, blocked and incubated with primary antibodies, anti-CD8 (1:5,000; 66868-1-Ig; Proteintech) or anti-PD-1 (1:5,000; 18106-1-AP; Proteintech), at 4°C overnight. These sections were then incubated with Alexa Fluor 488-conjugated goat anti-rabbit IgG(H+L) second antibody (1:1,000; AS053; ABclonal, Wuhan, China) or Alexa Fluor 555-conjugated goat anti-mouse IgG(H+L) second antibody (1:1,000; AS057; ABclonal). DAPI (28718-90-3; BBI Life Science, China) was used to stain the nuclei. The fluorescence images were acquired by Inverted Zeiss LSM880 laser scanning confocal microscope (Zeiss, Germany).

### Statistical analysis

GraphPad Prisms 7.0 software (GraphPad Software, San Diego, CA) was used for statistical analysis. Data were presented as means ± standard deviations (SD). For data comparisons between two groups, Student’s *t*-test was used; for data comparisons among groups more than two, one-way ANOVA test was used. The Pearson correlation analysis was utilized to analyze the correlations between variables. For survival analysis, log-rank test was performed. Chi-square test and Fisher exact test were used to assess the associations of the expression level of ST3GAL5 or the density of CD8^+^ and PD-1^+^ cell with clinicopathological parameters in ccRCC samples from our own cohort. The p value less than 0.05 was regarded statistically significant for all analyses.

## Results

### The mRNA levels of ST3GAL family members in ccRCC and normal kidney tissues

**Figure 1:**
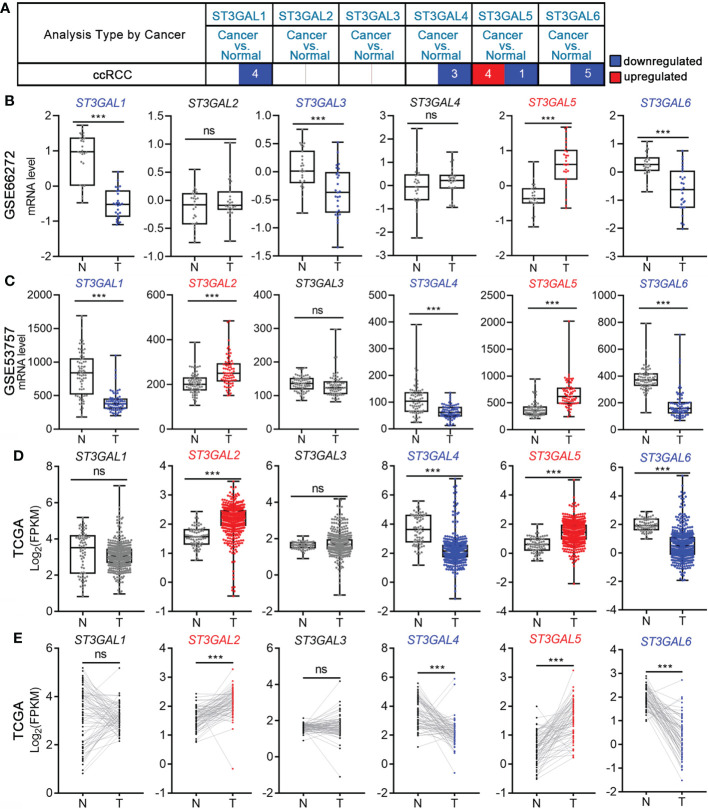
The transcription levels of six ST3GAL members in ccRCC and normal kidney tissues. **(A)** Differential mRNA expression analyses in ccRCC on Oncomine platform. The numbers of ccRCC datasets with significant different expression levels of ST3GAL family members were shown (blue, downregulated; red, upregulated). **(B-D)** Comparison of the mRNA expression levels of ST3GAL family members in ccRCCs (T) and normal kidneys (N) in GSE66272 (**B**; T = 27, N = 27), GSE53757 (**C**; T = 72, N = 72) and TCGA-KIRC (**D**; T = 530, N = 72). **(E)** Comparison of the mRNA expression levels of ST3GAL family members in paired normal (N) and ccRCCs (T) tissues (n=72). ***, p < 0.001; ns, p ≥ 0.05.

To explore the role of ST3GAL family members in ccRCC patients, we compared their mRNA levels between ccRCC and normal kidney tissues from Oncomine database. It was shown that the mRNA level of ST3GAL5 was significantly up-regulated in ccRCCs in four datasets and down-regulated in one dataset, compared with normal kidney tissues; while the levels of ST3GAL1, 4 and 6 were down-regulated in ccRCC tissues and no significant expression changes of ST3GAL2 and 3 were observed in ccRCC tissues (**Figure 1A**, **Table 1**). We then analyzed the mRNA levels of ST3GAL family members in another five ccRCC datasets (GSE66272, GSE53537, GSE36895, GSE105261 and TCGA-KIRC), containing 693 ccRCC samples and 203 normal kidney samples in total. Consistently, both upregulation of ST3GAL5 and downregulation of ST3GAL6 in ccRCC samples were identified in all five datasets (**Figures 1B–D** and **Supplementary Figure 1**). We noticed that ST3GAL5 upregulation (p < 0.001) and ST3GAL6 downregulation (p < 0.001) were confirmed in ccRCC tissues (n = 530), comparing with their paired normal kidney tissues (n = 72), from TCGA-KIRC dataset (**Figure 1E**).

**Table 1:** The mRNA levels of ST3GAL family members on Oncomine platform (ccRCC vs. normal kidney tissues).

Gene symbol	Type of ccRCC vs normal kidney tissue	ccRCC (n)	Normal (n)	Fold change	p value	Source and/or reference
ST3GAL1	ccRCC	23	23	-3.270	8.47E-19	Jones Renal Statistics (22)
	ccRCC	10	10	-3.206	0.001	Gumz Renal Statistics (23)
	Non-Hereditary ccRCC	27	11	-1.899	0.003	Beroukhim Renal Statistics (24)
	Hereditary ccRCC	32	11	-1.879	0.004	Beroukhim Renal Statistics
ST3GAL2	NA	NA	NA	NA	NA	NA
ST3GAL3	NA	NA	NA	NA	NA	NA
ST3GAL4	ccRCC	24	3	-3.681	0.005	Higgins Renal Statistics (25)
	ccRCC	10	10	-2.916	1.53E-5	Gumz Renal Statistics
	Hereditary ccRCC	32	11	-1.870	4.50E-5	Beroukhim Renal Statistics
ST3GAL5	ccRCC	22	3	1.797	0.016	Higgins Renal Statistics
	ccRCC	23	23	1.658	3.14E-11	Jones Renal Statistics
	ccRCC	26	5	2.280	4.25E-4	Yusenko Renal Statistics (26)
	ccRCC	10	10	-2.281	0.008	Gumz Renal Statistics
	Non-Hereditary ccRCC	27	11	2.009	4.79E-7	Beroukhim Renal Statistics
ST3GAL6	ccRCC	26	5	-1.621	0.023	Yusenko Renal Statistics
	ccRCC	23	23	-1.743	1.11E-4	Jones Renal Statistics
	ccRCC	10	10	-1.623	0.001	Gumz Renal Statistics
	Non-Hereditary ccRCC	27	11	-2.378	1.77E-8	Beroukhim Renal Statistics
	Hereditary Clear ccRCC	32	11	-2.118	5.61E-8	Beroukhim Renal Statistics

NA, Not Available.

### ST3GAL5 overexpression was positively associated with tumor development in ccRCC

**Figure 2:**
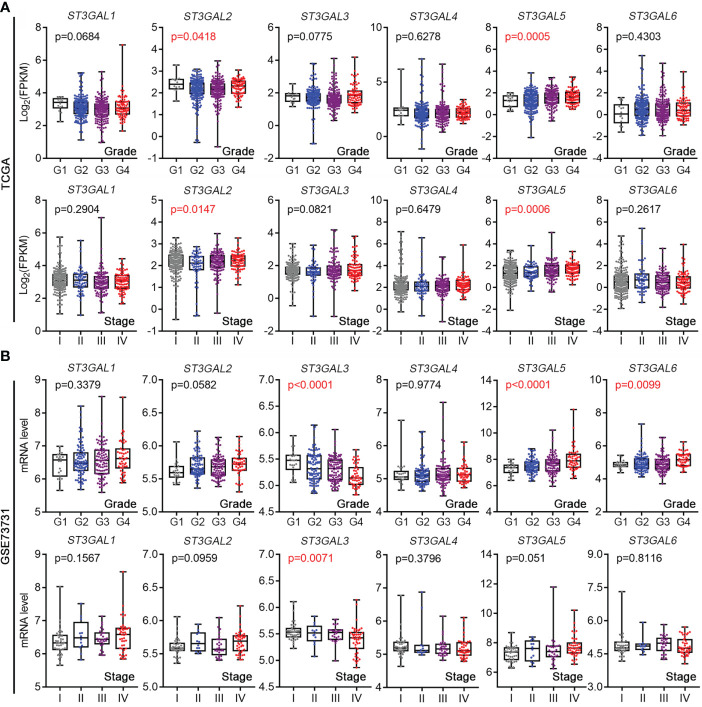
The expression levels of ST3GALs were correlated with the pathological stage and grade of ccRCC patients. **(A)** Correlations between mRNA expression levels of ST3GALs and tumor development in ccRCCs from TCGA-KIRC. Upper panel for tumor grade, Grade 1 = 14, Grade 2 = 227, Grade 3 = 206, Grade 4 = 75; Lower panel for tumor stage, Stage I = 265, Stage II = 57, Stage III = 123, Stage IV = 82. **(B)** Correlations between mRNA expression levels of ST3GALs and tumor development in ccRCCs from GSE73731. Upper panel for tumor grade, Grade 1 = 22, Grade 2 = 90, Grade 3 = 95, Grade 4 = 49; Lower panel for tumor stage, Stage I = 41, Stage II = 12, Stage III = 28, Stage IV = 44.

In order to study whether the expression of ST3GAL family members was related to tumor development in ccRCC, their mRNA levels were analyzed in TCGA-KIRC and GSE73731 datasets, which contain the corresponding clinicopathological information. In TCGA-KIRC dataset (n = 522 in total), ST3GAL5 mRNA expression was significantly elevated in higher grades ccRCC (G2-G4), compared with that in low grade (G1) (p = 0.0005; **Figure 2A** **, upper panel**). Overexpressed ST3GAL5 in higher stage ccRCC (II-IV) was also shown (p = 0.0006; **Figure 2A** **, lower panel**). However, ST3GAL6 expression did not exhibit significant correlations with tumor grade and tumor stage. In GSE73731 dataset (n = 256 in total), upregulated ST3GAL5 (p < 0.0001) and ST3GAL6 (p = 0.0099) was positively correlated only with tumor grade in ccRCC, respectively (**Figure 2B**). Altogether, these data indicated that ST3GAL5 overexpression might be positively associated with ccRCC development.

### Overexpressed ST3GAL5 was correlated with poor prognosis of ccRCC patients

**Figure 3:**
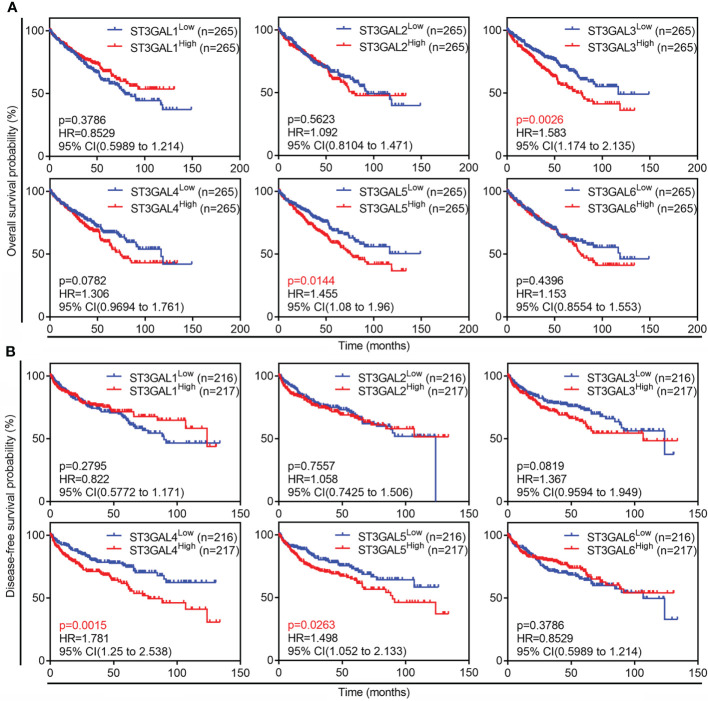
Prognostic values of six ST3GAL family members in ccRCCs. **(A)** and **(B)**, Kaplan-Meier plot of overall survival (**A**; n = 530) and disease-free survival (**B**; n = 433) in ccRCC patients from TCGA-KIRC dataset stratified by ST3GALs mRNA expression.

The correlation between expression of ST3GAL family members and outcome of ccRCC patients was then tested in TCGA-KIRC dataset. The patients with OS information (n = 530) were divided into two groups based on mRNA levels of ST3GAL members (median value as the cutoff). The Kaplan-Meier survival analysis showed that high level of ST3GAL3 and ST3GAL5 mRNA were inversely associated with OS (p=0.0026, HR=1.583 for ST3GAL3; p = 0.0144, HR = 1.455 for ST3GAL5; **Figure 3A**). The correlation between high level of ST3GAL4 and ST3GAL5 mRNA and short DFS time were also shown (p=0.0015, HR=1.781 for ST3GAL4; p = 0.0263, HR = 1.498 for ST3GAL5; **Figure 3B**) in the patients with DFS information (n = 433). The correlation between increased ST3GAL5 mRNA level and poor prognosis in ccRCC patients was suggested.

### ST3GAL5 might be involved in tumor immunoregulation

**Figure 4:**
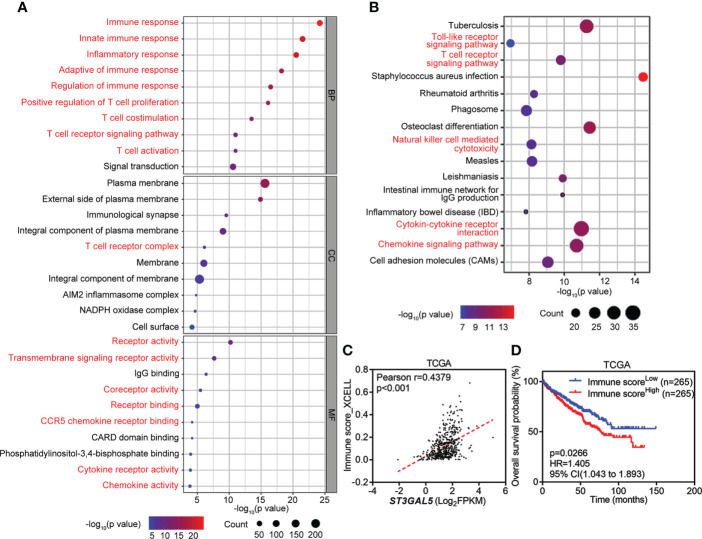
Involvement of *ST3GAL5* in tumor immunoregulation of ccRCC. **(A)** and **(B)** Gene Ontology (GO) enrichment analysis **(A)** and Kyoto Encyclopedia of Genes and Genomes **(KEGG)** analysis **(B)** of 645 genes co-expressed with *ST3GAL5* from TCGA-KIRC dataset. BP, biological processes; CC, cellular components; MF, molecular function. **(C)** The correlation between *ST3GAL5* mRNA expression and immune score by xCell tool in TCGA-KIRC dataset (n = 530). **(D)** Kaplan-Meier plot analysis of overall survival in ccRCC patients from TCGA-KIRC dataset stratified by xCell immune score.

To explore the regulatory network of ST3GAL5, 645 genes co-expressed with ST3GAL5 identified by Spearman correlation analysis using cBioPortal online tool (r ≥ 0.3, **Table S2**). GO molecular function enrichment analysis of these genes further revealed the prominent immune signatures, cytokine signatures and receptor signatures. T cell-relevant pathways, such as T cell co-stimulation, T cell proliferation, T cell activation, T cell receptor signaling pathway and T cell receptor complex, were especially noticed (**Figure 4A**). KEGG pathway analysis of these genes also showed the prominent presence of inflammation response signature (**Figure 4B**). Since xCell is a webtool that performs cell type enrichment analysis from gene expression data for 64 immune and stroma cell types, including T cell (37), we used it to calculate the immune score of ccRCC samples from TCGA-KIRC dataset (n = 530). The positive association between immune score and ST3GAL5 mRNA level expression in ccRCC patients was demonstrated by Pearson correlation analysis (r = 0.4379, p < 0.001) (**Figure 4C**). Consistent with the result in **Figure 3A**, high immune score was related to a poor OS by the Kaplan-Meier survival analysis (**Figure 4D**). The involvement of ST3GAL5 in tumor immunomodulation was thus indicated.

### ST3GAL5 was positively correlated with the infiltration of CD8^+^ T cells in ccRCC

**Figure 5:**
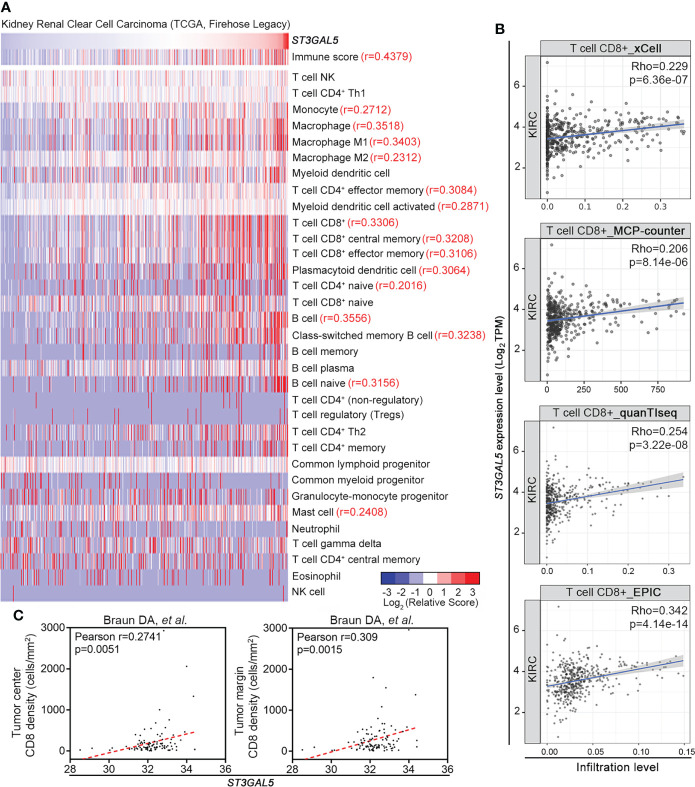
*ST3GAL5* was positively correlated with CD8^+^ T cell infiltration in ccRCC. **(A)** The correlations between *ST3GAL5* mRNA expression and infiltration fractions of different immune cells by xCell in TCGA-KIRC dataset using Pearson correlation analysis (n = 530). **(B)** The correlation between *ST3GAL5* mRNA expression and the infiltration level of CD8^+^ T cell by xCell, MCP-counter, quanTIseq and EPIC algorithms in ccRCC samples from TCGA-KIRC (n = 533). **(C)** The correlation between *ST3GAL5* mRNA expression and CD8^+^ T cells density in tumor center (left panel) and in tumor margin (right panel) in ccRCC sample from Braun DA cohort (32).

To dissect which type of immune cell populations might be associated with ST3GAL5 expression, xCell tool on TIMER2.0 web server was applied in TCGA-KIRC dataset (n = 530, **Figure 5A**). As expected, positive correlations of ST3GAL5 expression with immune cells were shown that 15 immune cell population (r ≥ 0.2, p < 0.05), including CD8^+^ T cells (r = 0.3208, p < 0.001), T cell CD8^+^ central memory (r = 0.3306, p < 0.001), T cell CD8^+^ effector memory (r = 0.3106, p < 0.001) and macrophage (r = 0.3518, p < 0.001). Since T cell-related signatures (**Figure 4A**) and pathways (**Figure 4B**) were enriched for ST3GAL5 overexpression in ccRCC patients, herein relationship between ST3GAL5 expression and tumor-infiltrated CD8^+^ T cells were further analyzed by TIMER2.0, a web server provides comprehensive analysis and visualization functions of tumor infiltrating immune cells (33). Positive correlations between ST3GAL5 mRNA level and infiltrating level of CD8^+^ T cells in TCGA-KIRC dataset were estimated significantly by four different algorithms, including xCell (Rho = 0.229, p < 0.001), MCP-counter (Rho = 0.206, p < 0.001), quanTIseq (Rho = 0.254, p < 0.001) and EPIC (Rho = 0.342, p < 0.001) (**Figure 5B**). Considering that Braun and colleague had assessed the cell density of CD8^+^ T cells in tumor center and margin of ccRCC by immunofluorescence staining, along with gene expression profiling of ccRCC samples by RNA-sequencing (32), we analyzed their above data and found that ST3GAL5 mRNA level was positively associated the density of both central and marginal CD8^+^ T cells in their ccRCC cohort (**Figure 5C**).

### Correlations between ST3GAL5 mRNA level and CD8^+^ T cell exhaustion in ccRCC

The common paradigm of CD8^+^ T cell infiltration is divided into three types, that is “immune infiltrated”, “immune excluded” and “immune desert” (38). In most solid tumors, high tumor-infiltrating CD8^+^ T cells (immune infiltrated) predict good clinical outcomes treated with PD-1 blockade, while immune exclusion or immune desert is associated with resistance to these therapies (38–40). However, our data showed that high expression of ST3GAL5 was associated with poor prognosis of ccRCC patients (**Figures 3A, B**) and enrichment of CD8^+^ T cells in ccRCC (**Figures 5A-C**). This “paradox” encouraged us to make further evaluation on the function of tumor-infiltrated CD8^+^ T cells.

In the Braun’s report (32), OS and PFS of ccRCC patients did not show significant difference between the “immune infiltrated” type, the “immune excluded” and “immune desert” types (p > 0.05). Moreover, the clinical benefit of anti-PD-1 treatment had no significant difference between the “immune infiltrated” type and the non-“immune infiltrated” types. Their results suggested that the complicated paradigm of CD8^+^ T cell infiltration might not improve the prognosis nor the clinical outcomes of PD-1 blockade in ccRCC patients. Whether other features of tumor-infiltrated CD8^+^ T cells could be used as the prognosis indicator of ccRCC was thus investigated.

Previous study reported that the effector function of tumor-infiltrating CD8^+^ T cells were impaired in ccRCC leading to the cell progression to exhaustion stage, which might account for tumor resistance to PD-1 blockade (4). These exhausted CD8^+^ T cells displayed persistent high expression of multiple inhibitory receptors, especially PD-1 (41). Therefore, we obtained the CD8^+^ T cell exhaustion profile using GSVA method (35), and analyzed its association with ST3GAL5 expression in ccRCC public datasets. The positive correlation between ST3GAL5 mRNA level and CD8^+^ T cell exhaustion signature score was shown in TCGA-KIRC (r = 0.3309, n = 530), GSE53757 (r = 0.4906, n = 72), and GSE73731 (r = 0.3397, n = 265), respectively (p<0.001 for all three datasets; **Supplementary Figure 2A**). Four “CD8^+^ T cell exhaustion”-associated genes by suppression T cell activity and promotion tumor immune escape were selected, including *PDCD1* gene which encodes PD-1 protein, *CD274* gene which encodes PD-L1 protein, *CTLA4* gene (an important exhaustion feature of lymphocyte (41)), and *CD86* gene whose encoding protein as the ligand of CTLA4 (42). Their associations with ST3GAL5 expression were further assessed. The positive correlations of mRNA levels between *ST3GAL5* and *PDCD1* (r = 0.3575, n = 530), *CD274* (r = 0.3018, n = 530), *CTLA4* (r = 0.39, n = 524) and *CD86* (r = 0.4127, n = 530) were identified in TCGA-KIRC dataset (p < 0.001 for all four genes; **Supplementary Figure 2B**). Similar results were also observed in GSE73731 dataset (p < 0.001 for all four genes, n = 265; **Supplementary Figure 2C**). These results from public datasets implied that ccRCC patients with ST3GAL5 overexpression might be prone to display CD8^+^ T cell exhaustion.

### ST3GAL5 overexpression was associated with the exhaustion of CD8^+^ T cells in ccRCC samples of our cohort

**Figure 6:**
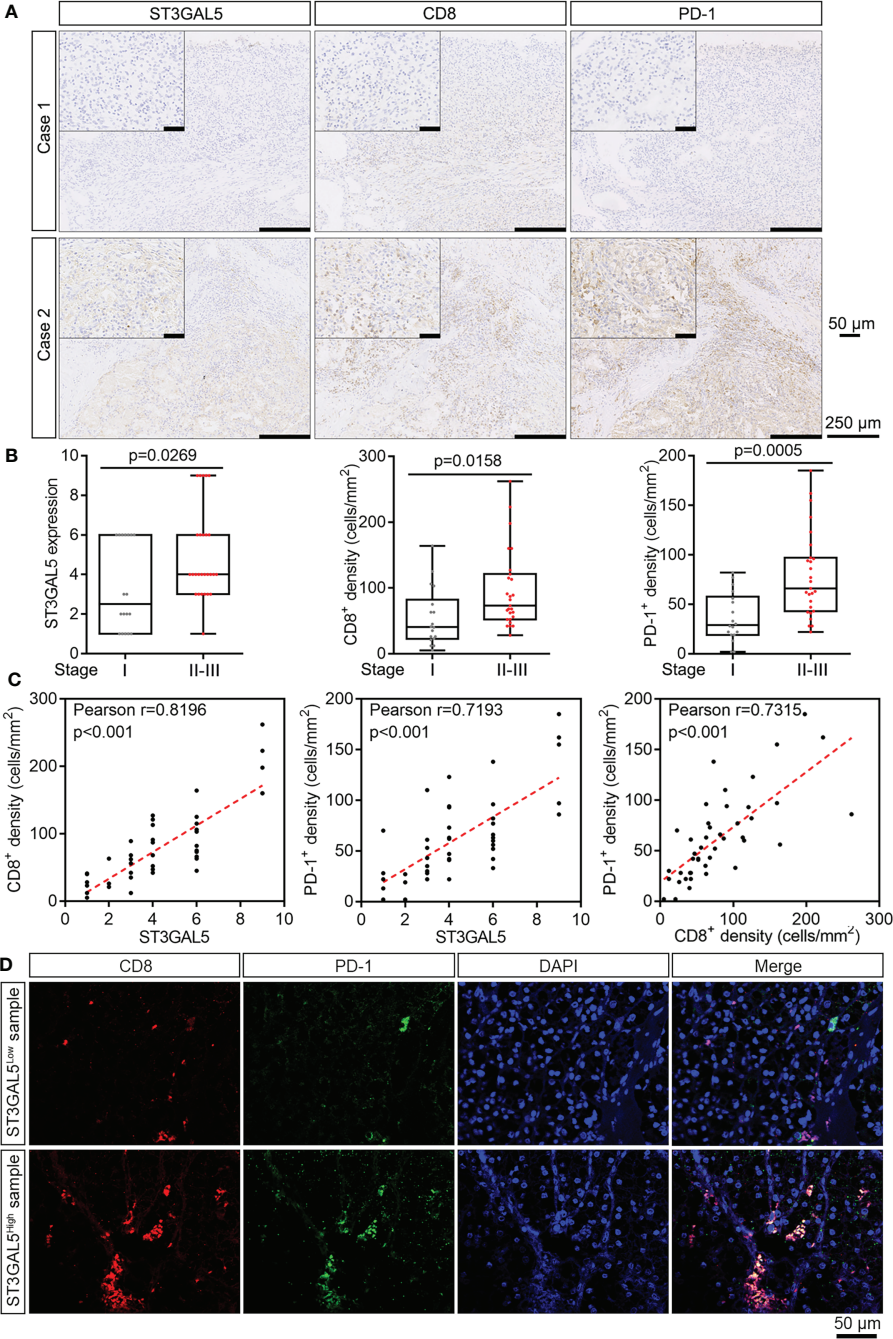
ST3GAL5 overexpression was associated with the exhaustion of CD8^+^ T cells in ccRCC samples of our cohort. **(A)** The representative IHC staining for ST3GAL5, CD8 and PD-1 in human ccRCC specimens. Case 1, low expression of ST3GAL5; Case 2, high expression of ST3GAL5. Scale bar, 250 μm; scale bar in inset, 50 μm. **(B)** Correlations between tumor stage and ST3GAL5 protein level (left panel), CD8^+^ cell density (middle panel), and PD-1^+^ cell density (right panel) in human ccRCC specimens (n = 45). Stage I, n = 18; Stage II-III, n = 27. **(C)** The correlations between ST3GAL5 level and CD8^+^ cell density (left panel), between ST3GAL5 level and PD-1^+^ cell density (middle panel), and between CD8^+^ and PD-1^+^ cell density (right panel), in human ccRCC specimens (n = 45). **(D)** Immunofluorescence co-staining of CD8 (red) and PD-1 (green) in human ccRCC specimens with low (upper panel) and high (lower panel) expression of ST3GAL5. DAPI (blue) was used for nuclei staining. Scale bar, 50 μm.

To validate above results from public datasets by multiple bioinformatics analyses, we collected 45 ccRCC samples with different tumor stages from Shanghai Pudong Hospital (**Table S3**). IHC staining for ST3GAL5, CD8, and PD-1 proteins were performed in these samples, with rabbit IgG and mouse IgG as negative controls (**Supplementary Figure 3A**). In addition, strong signals for ST3GAL5, CD8 and PD-1 were detected in cancer regions, while very weak and few positive signals for ST3GAL5, CD8 and PD-1 were observed in the adjacent normal kidney regions on the same field (**Supplementary Figure 3B**). Hence, these data above indicated that the IHC signals for ST3GAL5, CD8 and PD-1 were specific and reliable. Next, we found that the ccRCC patients with high tumor stage (Stage II and III) (case 2 in **Figure 6A**) showed increased protein levels of ST3GAL5, CD8 and PD-1, compared to those with low tumor stage (Stage I) (case 1 in **Figure 6A**). Of note, CD8^+^ T cells were significantly enriched in both center and margin of tumors with ST3GAL5 high expression in our cohort (**Figure 6A**), which was consistent with that in Braun’s cohort (**Figure 5C**). Quantification of these proteins was then performed for ST3GAL5 IHC score, CD8^+^ cell density and PD-1^+^ cell density. It was shown that the elevated IHC score for ST3GAL5 and the densities of CD8^+^ and PD-1^+^ cells were positively correlated with high tumor stage, respectively (p < 0.05; **Figure 6B**). Furthermore, the protein expression levels of ST3GAL5 and the density of CD8^+^ cells and PD-1^+^ cells were positively correlated with each other in ccRCC tissues (r > 0.7, p < 0.001; **Figure 6C**), suggesting the presence and exhaustion of CD8^+^ T cells in ccRCC samples with high stage. IF co-staining for CD8 and PD-1 was thus performed in ccRCC samples with low and high expression of ST3GAL5, respectively. In ST3GAL5^high^ samples, fluorescence intensities for CD8 and PD-1 were much higher than those in ST3GAL5^low^ samples. Remarkable increased co-localization of CD8 and PD-1 proteins were observed in ST3GAL5^high^ samples (**Figure 6D**), indicating the positive association between ST3GAL5 expression and exhausted CD8^+^ T cells in ccRCC tissue.

In conclusion, we have demonstrated that ST3GAL5 could be used as a prognostic biomarker for ccRCC, predicting the effector function of CD8^+^ T cells.

## Discussion

Aberrant sialylation on tumor cell surface and hyperactivity of sialyltransferases are common characteristics of cancers, often taken as a hallmark in many cancers (9). In our study, we comprehensively analyzed the expression and prognostic relevance of ST3GAL family members in multiple independent ccRCC datasets, revealing that ST3GAL5 could be a potential prognostic biomarker. High expression of ST3GAL5 predicted poor clinical outcomes in ccRCC patients. Moreover, ST3GAL5 expression was positively associated with immune infiltration and CD8^+^ T cell exhaustion.

ST3GAL5 functions as a synthase for glycosphingolipid GM3, a precursor of a- and b- and c- series gangliosides, which participates in various cellular processes, such as cell proliferation and differentiation, and integrin-mediated cell adhesion (43, 44). In line with our aforementioned findings in ccRCCs, the increase of ST3GAL5 expression was observed in CD44^hi^/CD24^lo^ breast cancer stem-like cells (CSCs), compared with non-CSC population (45), while the enzymatic activity of ST3GAL5 is required for TGF-β1-induced ZEB1-dependent epithelial-to-mesenchymal transition (46). On the contrary, the metabolite GM3 can interfere with EGFR dimerization and suppress its activity through its binding with N-glycans of EGFR (47). Hence, it is not surprising that the downregulation of ST3GAL5 was reported in bladder cancer (48). Its downregulation can also promote radioresistance of melanoma cells by the activation of MAPK signaling and chemoresistance of acute myeloid leukemia *via* the activation of PI3K/AKT signaling (49). Overall, the role of ST3GAL5 in carcinogenesis is cancer-type dependent.

In addition, it was also reported that ST3GAL5 was expressed in non-epithelia cells, such as immune cells and adipose stromovascular cells (50, 51). Considering the infiltrating immune cells in the tumor areas, we could not entirely exclude the contribution of the ST3GAL5 expression in non-tumor cells to the conclusion on its overexpression in the ccRCCs by the transcriptomic data in TCGA-BLCA and GEO datasets alone. To address this concern, we carried out IHC assays and our data clearly demonstrated that ccRCC cells displayed higher ST3GAL5 protein expression levels than adjacent normal epithelial cells in the same section. Taken together, in future it is necessary to characterize the roles of ST3GAL5 in cancer cells and nonmalignant cells in the tumor stroma during ccRCC development.

Our study further revealed that ST3GAL5 overexpression in ccRCC is significantly associated with immune infiltration (including CD8^+^ T cells, macrophages and B cells) and CD8^+^ T cell exhaustion, suggesting its overexpression may influence tumor immune microenvironment. Usually the tumor-infiltrated CD8^+^ T cells fully or partly lose their effector function because of the progression to a stage of exhaustion (41), which were frequently detected in many human cancers (4, 35). The exhausted CD8^+^ T cells show the persistent high expression of multiple inhibitory receptors (such as PD-1 and CTLA4), transcriptional and epigenetic reprogramming, metabolic dysregulation, and loss of effector function (41). Though immunotherapy *via* blocking PD-1/PD-L1 interaction is usually effective to partially reinvigorate the CD8^+^ T cells inside tumor, unfortunately, in advanced ccRCCs there were no association between CD8^+^ T cell infiltration and clinical benefit to anti-PD-1 therapy (32, 52, 53). Previous study reported that tumor-derived gangliosides suppress the cytotoxicity of CD8^+^ T cells by impeding TCR-induced lytic granule release (54). In addition to CD8^+^ T cells, the infiltrating macrophages and B cells inside tumors were also significantly associated with ST3GAL5 levels in cancer cells. Previous study reports a co-evolution between CD8^+^ T cells exhaustion and tumor-associated macrophage, *i.e.* tumor-associated macrophages promote the progression of CD8^+^ T cell exhaustion by long-lasting synapses with CD8^+^ T cells, and in return the exhausted CD8^+^ T cells shape myeloid cell recruitment and contribute to tumor-associated macrophage maturation (55). As for B cells, it is one of the major tumor-infiltrating immune cells in several solid tumors. However, the function of B cells on tumor development is controversial. On one hand, B cells possess the tumor suppressive effects on the promotion of T cell response, and on the other hand, B cells promote the tumor progression by secreting immunosuppressive cytokines (56). A recent study identified three B cell subpopulations in ccRCC biopsies and a B2M subpopulation played an essential pro-metastatic role in ccRCCs (57). Therefore, in future functional assays are needed to investigate whether the enzymatic activity of ST3GAL5 and its metabolite GM3 are indispensable for T cell exhaustion, as well as other immune cells. Overall, further studies on how ST3GAL5 modulates the pathogenesis of ccRCC and CD8^+^ T cells exhaustion are expected.

## Conclusion

In summary, our data indicated that ST3GAL5 could be a potential prognostic biomarker for clinical outcomes and might be a potential indicator of CD8^+^ T cell exhaustion in ccRCC patients.

## Data availability statement

The datasets presented in this study can be found in online repositories. The names of the repository/repositories and accession number(s) can be found in the article/**Supplementary Material**.

## Ethics statement

The studies involving human participants were reviewed and approved by Ethics Committee of Shanghai Pudong Hospital. The patients/participants provided their written informed consent to participate in this study.

## Author contributions

JY, RZ and RH conceived the project. JL, JW, SW, QQ, and ZZ performed the data acquisition. JL and JW performed the data assembly and analysis. YL, YZ and SL were responsible for collecting clinical samples and patient information. ML contributed to analyze the clinical samples by IHC and IF. JL, ML, JW, JY and RH contributed to the data interpretation and manuscript writing. All authors reviewed and approved the final manuscript.

## Funding

This work was supported by the National Natural Science Foundation of China (82172001 to RH and 82103415 to JL), Shanghai Municipal Science and Technology Major Project and the fund from Shanghai Science and Technology Committee (Grant number 20S11901400 to RH), Shanghai Municipal Health Commission Project (Grant No. 202140267 to RZ), Talents Training Program of Pudong Hospital affiliated to Fudan University (Grant No. PY202001 to RZ), the Project of Key Medical Discipline of Pudong Hospital of Fudan University (Grant No. Zdxk2020-04 to RZ), the Project of Key Medical Specialty and Treatment Center of Pudong Hospital of Fudan University (Grant No. Zdzk2020-01 to RZ).

## Acknowledgments

We thank Xiao He and the Institutional Center for Shared Technologies and Facilities of Shanghai Institute of Materia Medica, Chinese Academy of Sciences for technical supports.

## Conflict of interest

The authors declare that the research was conducted in the absence of any commercial or financial relationships that could be construed as a potential conflict of interest.

## Publisher’s note

All claims expressed in this article are solely those of the authors and do not necessarily represent those of their affiliated organizations, or those of the publisher, the editors and the reviewers. Any product that may be evaluated in this article, or claim that may be made by its manufacturer, is not guaranteed or endorsed by the publisher.

## Abbreviations

BP, Biological Processes; CC, Cellular Components; ccRCC, Clear Cell Renal Cell Carcinoma; CI, Confidence Interval; CSCs, Cancer Stem-like Cells; CTLA4, Cytotoxic T-lymphocyte Associated protein 4; DFS, Disease-Free Survival; EGFR, Epidermal Growth Factor Receptor; G 1-4, grade 1-4; GO; Gene Ontology; GSVA, Gene Set Variation Analysis; HR, Hazard Ratio; IF, Immunofluorescence; IHC, Immunohistochemistry; KEGG, Kyoto Encyclopedia of Genes and Genomes; KIRC, Kidney Renal Clear Cell Carcinoma; MAPK, Mitogen Activated Kinase-Like Protein; MF, Molecular Function; OS, Overall Survival; PDCD1/PD-1, Programmed Cell Death 1; PD-L1, Programmed Cell Death 1 Ligand 1; ST3GAL1-6, ST3 beta-galactoside alpha-2,3-sialyltransferase 1-6; TCGA, The Cancer Genome Atlas; TCR, T Cell Receptor; TGF-β1, Transforming Growth Factor-β1; ZEB1, Zinc Finger E-box Binding Homeobox 1.
