# Supplementary material for: Identification of ST3GAL5 as a prognostic biomarker correlating with CD8+ T cell exhaustion in clear cell renal cell carcinoma
Source: Front Immunol. 2022 Sep 12;13:979605. doi: 10.3389/fimmu.2022.979605 (PMC9510991; doi:10.3389/fimmu.2022.979605)
Supplement: Supplementary file 1 [file DataSheet_1.docx]

Supplementary Material

**Identification of ST3GAL5 as a prognostic biomarker correlating with CD8^+^ T cell exhaustion in clear cell renal cell carcinoma**

**Jiakuan Liu^1,2, †^, Meiqian Li^1,3, †^, Jiajun Wu^2, †^, Qi Qi^2^, Yang Li^1^, Simei Wang^4,5^, Shengjie Liang^1^, Yuqing Zhang^1^, Zhitao Zhu^2^, Ruimin Huang^4,5,*^, Jun Yan^1,2,*^ and Rujian Zhu^1,*^**

^1^ Department of Urology, Shanghai Pudong Hospital, Fudan University Pudong Medical Center, Shanghai 201399, China.

^2^ Department of Laboratory Animal Science, Fudan University, Shanghai 200032, China.

^3^ Model Animal Research Center of Nanjing University, Nanjing University, Jiangsu 210061, China.

^4^ Shanghai Institute of Materia Medica, Chinese Academy of Sciences, Shanghai 201203, China.

^5^ University of Chinese Academy of Sciences, Beijing 100049, China.

^†^ These authors have contributed equally to this work and shared the first authorship.

***Correspondence:**

Ruimin Huang

rmhuang@simm.ac.cn

Jun Yan

yan_jun@fudan.edu.cn

Rujian Zhu

20667@shpdh.org


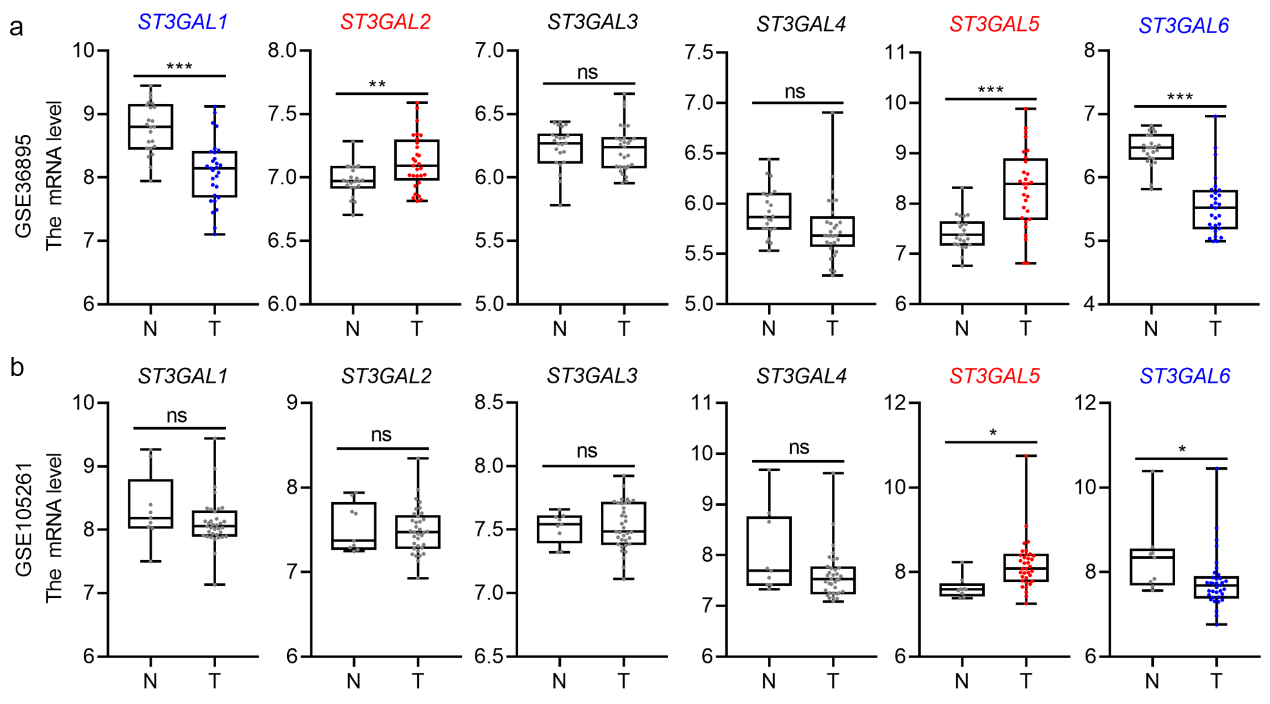


**Supplementary Figure 1.** The transcription levels of six ST3GAL members in ccRCC and normal kidney specimens. **(A)** and **(B)** Comparison of the expression levels of ST3GAL family members in normal kidney tissue (N) and ccRCC (T) in GSE36895 **(A)** and GSE105261 **(B)** datasets. GSE36895-Normal (n=23), GSE36895-Tumor (n=29); GSE105261-Normal (n=9), GSE105261-Tumor (n=35). ***p< 0.001, **p< 0.01, *p< 0.05; ns, no significant difference.

**
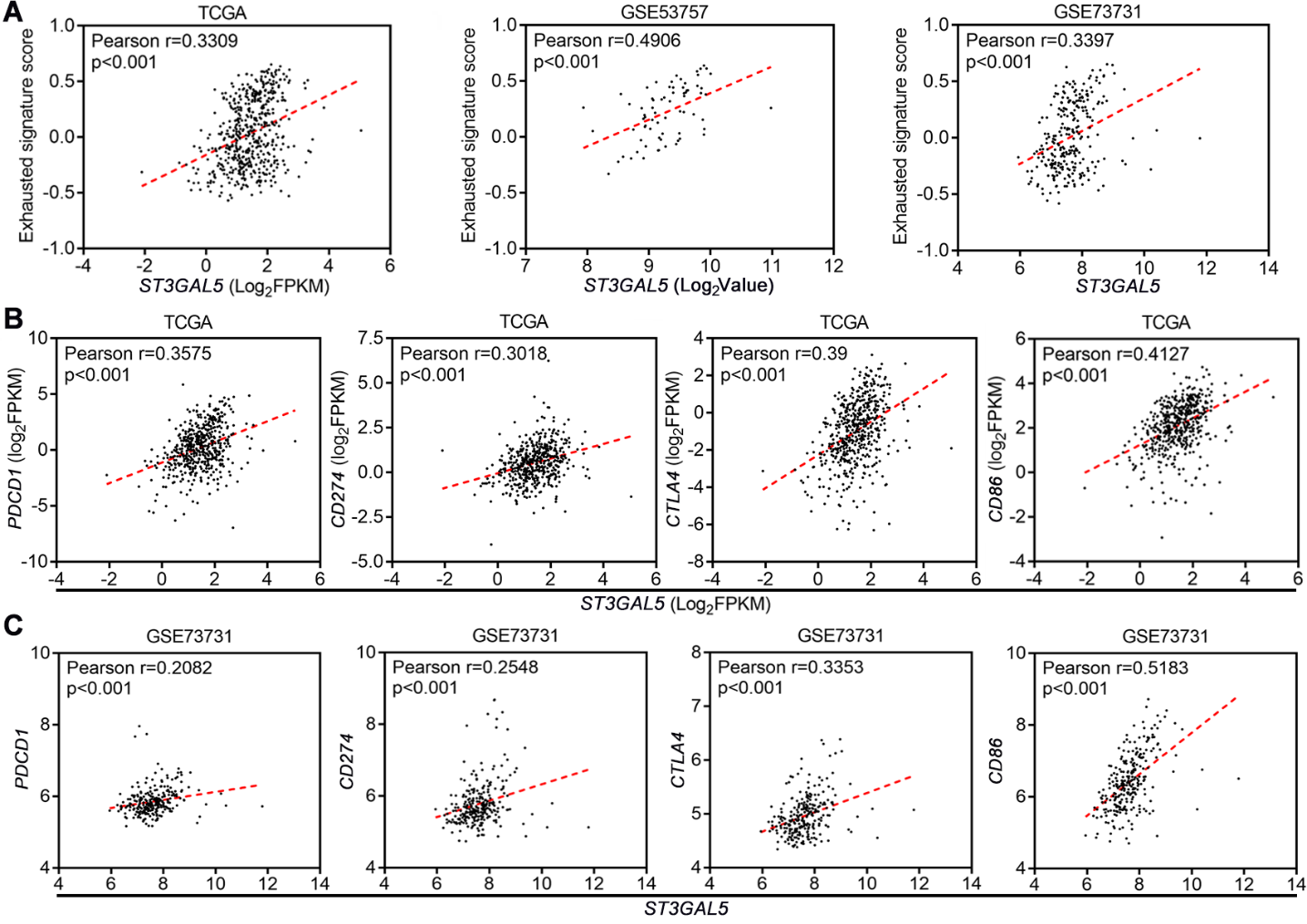
**

**Supplementary Figure 2**. The correlation between *ST3GAL5* mRNA expression and CD8^+^ T exhaustion in ccRCC. **(A)** The correlation between *ST3GAL5* mRNA expression and CD8^+^ T cells exhaustion signature score in TCGA-KIRC (left; n = 530), GSE53757 (middle; n = 72) and GSE73731 (right; n = 265) datasets. **(B)** The correlations of mRNA expression level between ST3GAL5 and CD8^+^ T cell exhaustion-associated genes, including *PDCD1* (n = 530), *CD274* (n = 530), *CTLA4* (n = 524), and *CD86* (n = 530), in TCGA-KIRC dataset. **(C)** The correlations of mRNA expression levels between S*T3GAL5* and CD8^+^ T cell exhaustion-associated genes, including *PDCD1*, *CTLA4*, *CD274*, and *CD86*, in GSE73731 dataset (n = 265).

**
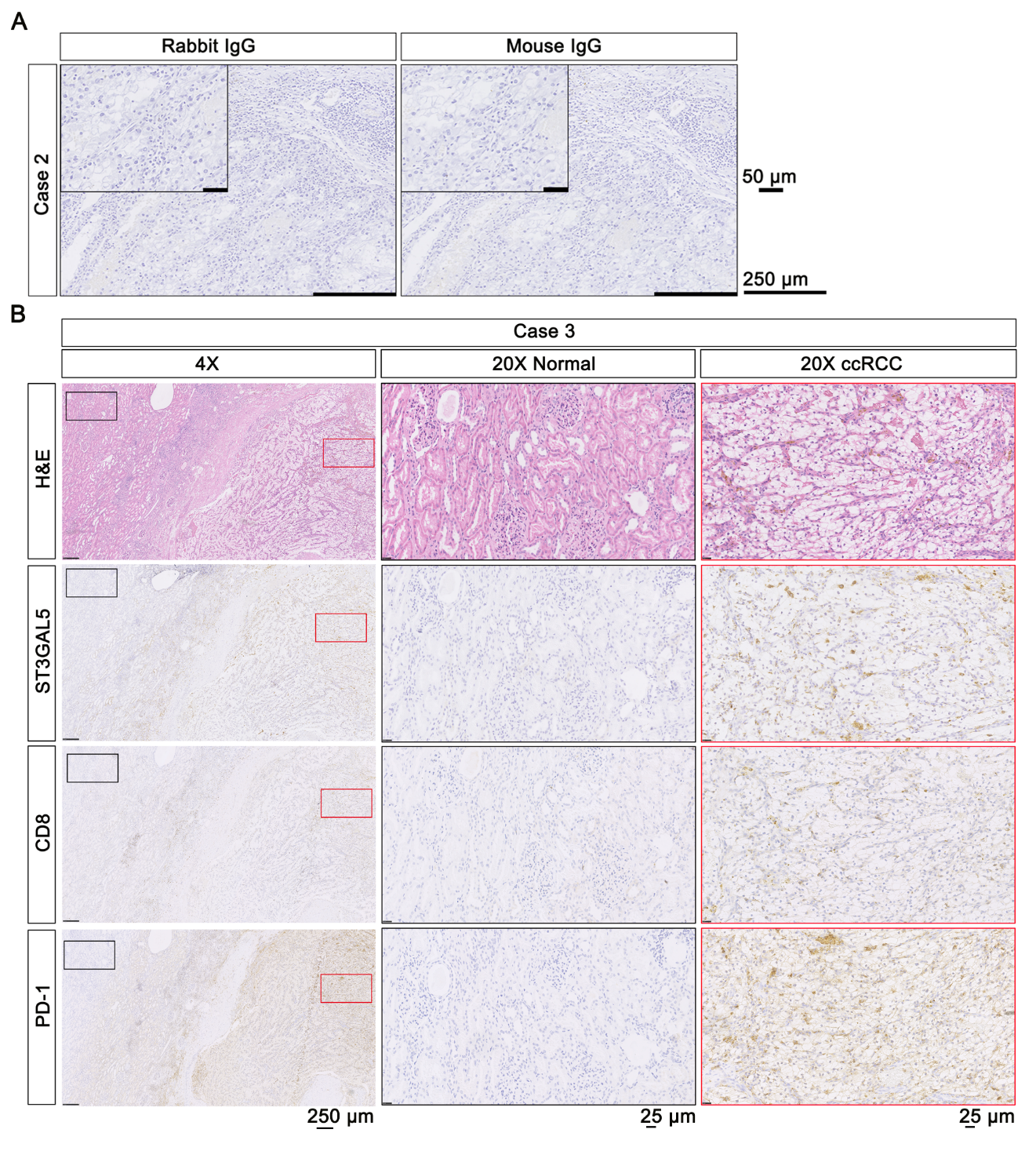
**

**Supplementary Figure 3.** **(A)** IHC staining for IgG controls. The rabbit IgG **(left)** and the mouse IgG **(right)** were used for IHC staining on sections from Case 2, which were served as negative controls. Scale bar, 250 μm; scale bar in insets, 50 μm. **(B)** The IHC staining for ST3GAL5, CD8 and PD-1 on adjacent ccRCC sections from Case 3. Black boxes, the normal kidney regions; red boxes, ccRCC regions. Scale bar (left panel), 250 μm; scale bar (middle and right panels), 50 μm.

**Table S1.** The gene list for the exhausted T cell signature

**Table S2.** 645 genes co-expressed with ST3GAL5 identified by spearman's correlation analysis using cBioPortal online tool

**Table S3.** The correlation between the ST3GAL5 protein expression, the density of CD8^+^ or PD-1^+^ cells and clinicopathological parameters of 45 ccRCC samples from our IHC cohort
